# Emergency Embolization of Hemorrhagic Greater Superficial Petrosal Nerve (GSPN) Schwannoma: A Rare Case of Intracranial Hemorrhage

**DOI:** 10.7759/cureus.89119

**Published:** 2025-07-31

**Authors:** Yugandhar S, Rajasekhar Rekapalli, Vivek Lanka, Kavya Dasari, Komala Sai Phanindra Edala

**Affiliations:** 1 Radiodiagnosis, All India Institute of Medical Sciences, Mangalagiri, IND; 2 Neurological Surgery, All India Institute of Medical Sciences, Mangalagiri, IND; 3 Neuroimaging and Interventional Radiology, Krishna Institute of Medical Sciences (KIMS) Sikhara, Guntur, IND

**Keywords:** endovascular embolization, greater superficial petrosal nerve, middle meningeal artery, petrous branch, schwannoma, subdural hematoma

## Abstract

Greater superficial petrosal nerve (GSPN) schwannomas are exceptionally rare and have not been previously reported to present with acute hemorrhage. In contrast, hemorrhagic vestibular and trochlear schwannomas have been described, often presenting with abrupt neurological symptoms. We report a case of a 41-year-old woman who presented with a sudden-onset headache and vomiting. CT revealed a hyperdense extra-dural lesion in the right middle cranial fossa, arising from an enlarged GSPN hiatus, with a subdural hematoma along the tentorium and fronto-parieto-temporal convexity (maximum thickness: 5 mm). MRI demonstrated a 25 × 20 mm extra-axial mass with heterogeneous T2 signal and perilesional edema. CT angiography showed intense enhancement, and digital subtraction angiography revealed a robust tumor blush with active contrast extravasation from the petrous branch of the middle meningeal artery. Super-selective embolization using 150-250 μm polyvinyl alcohol (PVA) particles achieved complete devascularization. Histopathological confirmation was not obtained, as the patient opted for conservative management; diagnosis was based on characteristic imaging features and precise anatomical localization. Post-embolization, the patient’s symptoms resolved, and she was discharged neurologically intact by day 7. She remained stable at the three-month follow-up without symptom recurrence. This case represents the first reported instance of a hemorrhagic GSPN schwannoma. It underscores the importance of considering rare bleeding neural tumors in acute headache presentations and demonstrates that trans-arterial embolization can offer immediate hemostasis and clinical stabilization, providing a critical window for safe transition to planned surgical excision. This strategy may serve as a valuable adjunct in the management of similar cases of other hypervascular cranial nerve tumors presenting with acute hemorrhage.

## Introduction

Schwannomas arising from the greater superficial petrosal nerve (GSPN) are exceedingly rare, accounting for less than 2% of all intracranial schwannomas [1]. Fewer than 40 cases have been reported in the literature, most presenting insidiously with symptoms such as facial dysesthesia, xerophthalmia, or conductive hearing loss [2]. To date, spontaneous hemorrhage from a GSPN schwannoma has not been reported.

In contrast, hemorrhagic transformation has been described in schwannomas of other cranial nerves, particularly the trochlear and vestibular nerves, occasionally presenting with acute headache, neurologic deficits, or rapid clinical deterioration [3,4]. Rare instances of hemorrhage have also been reported in vagal schwannomas, with presentations ranging from brainstem compression to apnea [5]. Proposed mechanisms include rupture of fragile intratumoral vessels, spontaneous thrombosis and necrosis, or venous congestion-although robust pathophysiological evidence remains limited [3,5].

The GSPN arises from the geniculate ganglion of the facial nerve and traverses the petrosal canal to enter the middle cranial fossa, coursing anteromedially toward the foramen lacerum. It carries parasympathetic fibers destined for the lacrimal gland and mucosa of the nasal cavity and palate via the pterygopalatine ganglion. Its location along the anterior superior surface of the petrous temporal bone makes it a potential site for skull base lesions, though schwannomas originating here are extremely uncommon [6].

The differential diagnosis for a hemorrhagic, enhancing extra-axial lesion in the temporal region includes meningioma with intratumoral hemorrhage, hemorrhagic metastasis (e.g., melanoma and choriocarcinoma), cavernous hemangioma, and dural-based hemangiopericytoma [7-10]. Recognition of characteristic imaging features-such as a lesion arising from the widened GSPN hiatus, following the anatomic course of the facial nerve-can help distinguish GSPN schwannomas from these entities.

We report the first known case of a hemorrhagic GSPN schwannoma, presenting with subdural hemorrhage and managed successfully with trans-arterial embolization (TAE). This case expands the spectrum of hemorrhagic cranial nerve schwannomas and highlights the potential of TAE as a stabilizing intervention in such emergent neurovascular scenarios.

## Case presentation

A 41-year-old right-handed woman with no medical comorbidities experienced a sudden-onset right temporal headache while at rest, followed by projectile vomiting and photophobia. She denied head trauma or antithrombotic use but was complaining of a recent onset of right earache for the past few months. On arrival to the emergency department, her Glasgow Coma Scale score was 15/15; pupils were equal and reactive; no facial nerve or ocular-motor deficits were noted. Laboratory tests, including coagulation profile, were normal.

**Figure 1:**
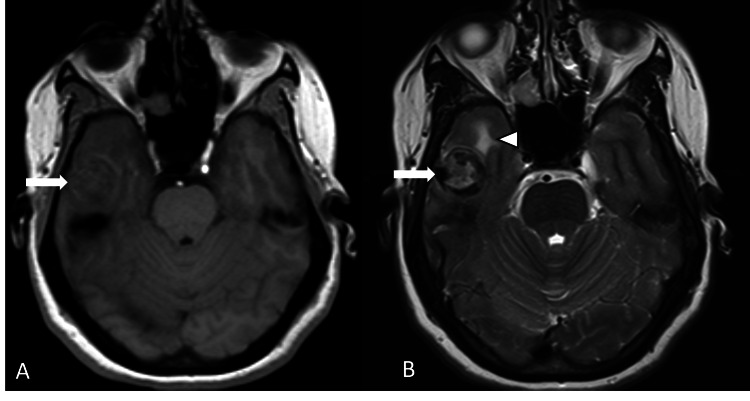
(A, B) Axial T1 and T2W images demonstrating a well-defined T1 isointense and T2 heterogeneous signal intensity lesion along the right temporal convexity (arrows). There is surrounding T2 hyperintense perilesional edema in the adjacent right temporal lobe white matter (arrowhead).

MRI brain revealed a well-circumscribed T1 isointense and T2 heterogeneously hyperintense hemorrhagic extra-axial mass lesion measuring approximately 25 × 20 mm in the right middle cranial fossa with perilesional edema in the adjacent temporal lobe white matter (Figures 1A, 1B).

**Figure 2:**
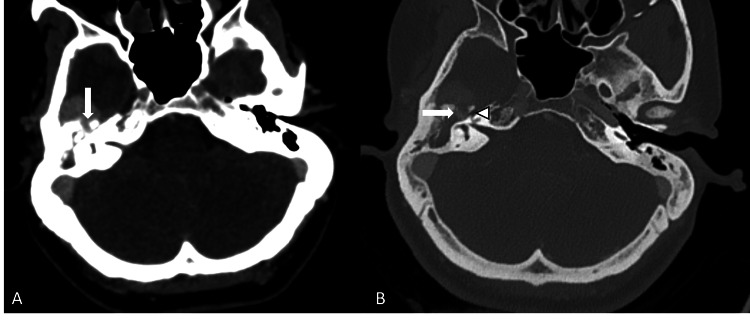
(A) Axial CECT image demonstrating a tongue-like projection of the enhancing tumor into the right petrous bone (arrow). (B) Axial HRCT image demonstrating dilated hiatus for the greater petrosal nerve (arrow). The genu of the facial nerve is marked by an arrowhead. CECT: contrast-enhanced CT; HRCT: high-resolution CT

The lesion demonstrated a tongue-like extension into the right petrous bone through an enlarged hiatus for the GSPN. High-resolution CT delineated osseous remodeling with widening of the GSPN hiatus, suggestive of a GSPN schwannoma (Figures 2A, 2B).

**Figure 3:**
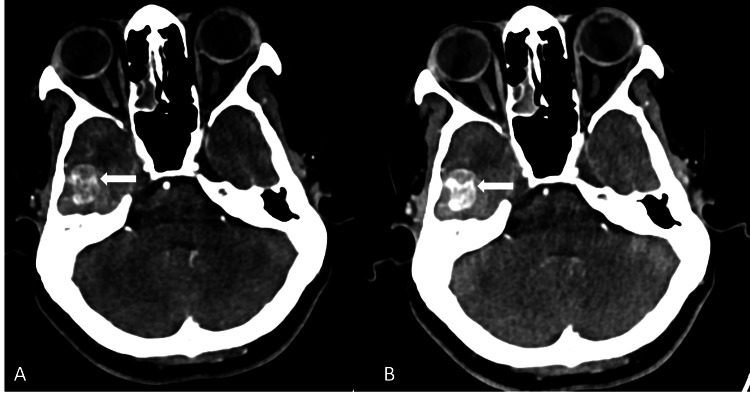
(A, B) Axial contrast-enhanced CT images in arterial and venous phases demonstrate intense heterogeneous enhancement of the right temporal extra-axial lesion with progressive increase in enhancement in the venous phase (arrows).

Contrast-enhanced CT in arterial and venous phases demonstrated intense heterogeneous enhancement of the lesion, with progressive accumulation of contrast on delayed imaging, suggesting intralesional hemorrhage (Figures 3A, 3B).

**Figure 4:**
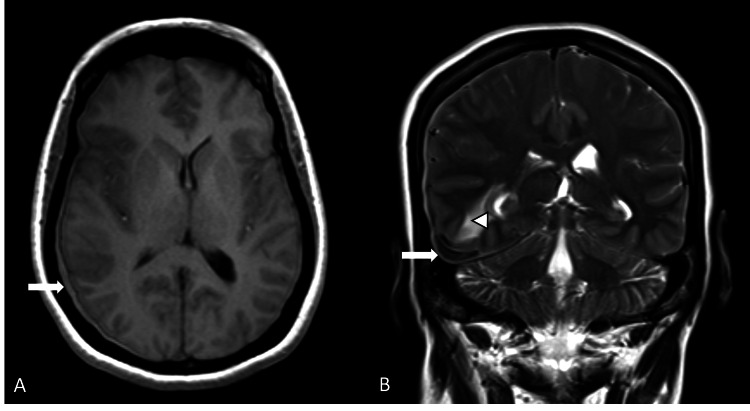
(A, B) Axial T1W image and coronal T2W images demonstrate thin T1 isointense and T2 hypo- to hyperintense subdural hematoma along the right fronto-parieto-temporal convexity (arrows). There is T2 hyperintense perilesional edema in the right temporal lobe white matter (arrowhead).

A thin, acute subdural hematoma was noted tracking along the right fronto-parieto-temporal convexity and tentorium, with a maximum thickness of 5 mm and no evidence of midline shift (Figures 4A, 4B).

**Figure 5:**
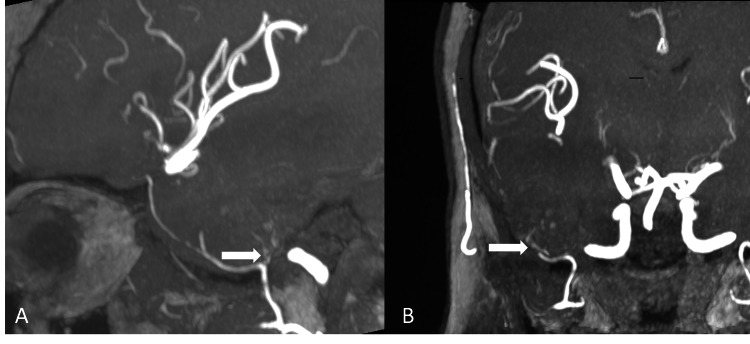
(A, B) Sagittal and coronal maximum intensity projection (MIP) images of time-of-flight (TOF) MR angiogram demonstrating the lesion supplied by the petrous branch of the middle meningeal artery (arrows).

Time-of-flight MR angiography demonstrated a serpiginous feeding vessel arising from the petrous branch of the middle meningeal artery (MMA), confirming vascular supply to the lesion (Figures 5A, 5B).

**Figure 6:**
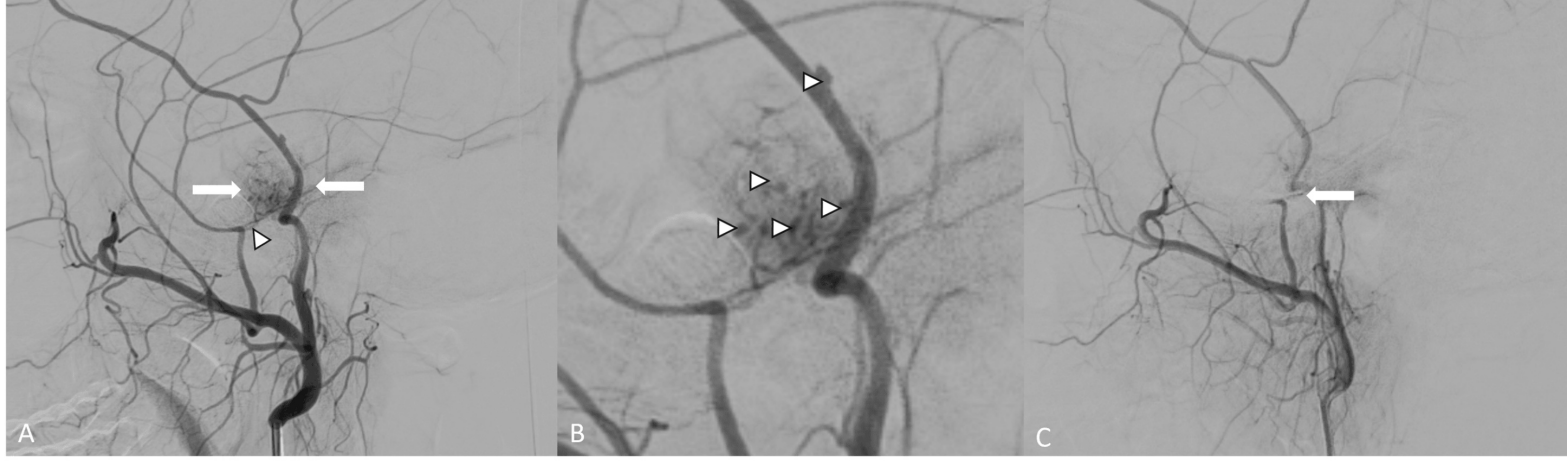
(A) Angiogram from the right STA-MMA common trunk demonstrating tumor blush (arrows) supplied by the petrous branch of MMA (arrowhead). (B) Magnified view of the tumor blush demonstrating multiple tiny points of contrast extravasation (arrowheads). (C) Post-embolization angiogram demonstrating complete exclusion of the tumor blush with contrast stasis in the petrous branch of MMA (arrows). STA: superficial temporal artery; MMA: middle meningeal artery

Suspected active bleeding from the lesion prompted urgent diagnostic angiographic evaluation. The patient underwent continuous vital monitoring during the procedure, including non-invasive blood pressure, oxygen saturation, and cardiac rhythm. Conscious sedation was administered with an anesthesiology standby. Microcatheter stability and flow control were confirmed with test injections before embolization. Right radial artery access was taken, and a 5 F Sim 2 glide catheter was navigated into the right common carotid artery (CCA) with its tip positioned in the right external carotid artery. Selective angiogram demonstrated a dense tumor blush supplied exclusively by the petrous branch of the MMA with multiple punctate foci of extravasation (Figures 6A, 6B). A 1.98 F microcatheter was navigated over a 0.014-inch microwire into the distal petrous branch. After confirming microcatheter stability, 150-250 µm polyvinyl alcohol (PVA) particles were infused slowly until near-stasis was achieved. Control angiography showed complete obliteration of the tumor blush without residual extravasation (Figure 6C).

Adjunctive corticosteroid therapy was administered post-procedure to mitigate vasogenic edema seen on pre-procedure imaging and to control perilesional inflammation. Hemodynamic parameters were stable throughout, and no periprocedural complications occurred. The patient demonstrated clinical improvement within 48 hours, and follow-up CT confirmed stabilization of the subdural hematoma without further hemorrhagic extension. Given this favorable response and the patient’s preference, surgical excision was deferred, with plans for continued neurosurgical and radiological follow-up. Three months post-discharge, the patient reported no significant headache and remained clinically stable (Table 1).

**Table 1:** Concise clinical summary. GCS: Glasgow Coma Scale; TOF-MRA: time-of-flight MR angiogram; MMA: middle meningeal artery; PVA: polyvinyl alcohol

Parameter	Findings
Age/sex	41-year-old female
Duration of symptoms	Right earache for several months; acute headache and vomiting on the day of presentation
Neurological status (on arrival)	GCS 15/15; no focal neurological deficits
Coagulation profile	Normal
Initial imaging	MRI: extra-axial hemorrhagic lesion in right middle cranial fossa with perilesional edema; CT: dilated hiatus of greater superficial petrosal nerve (GSPN); subdural hematoma (5 mm) TOF-MRA: serpiginous feeder from petrous branch of middle meningeal artery
Angiographic findings	Tumor blush with multiple punctate extravasations from the petrous branch of MMA
Embolization details	Trans-radial access; 5 F Sim 2 catheter; 1.98 F microcatheter; 150–250 µm PVA particles used
Periprocedural monitoring	Conscious sedation; non-invasive hemodynamic monitoring; anesthesia team on standby
Post-procedure therapy	Corticosteroids administered to address perilesional edema
Clinical course	Headache improved post-embolization; stable subdural hematoma on follow-up CT
Definitive surgical plan	Surgical resection discussed; patient declined; continued neurosurgical follow-up advised

## Discussion

Literature review

Since the initial description of GSPN schwannoma in 1962, only isolated cases have been reported in the literature [1]. To our knowledge, a hyperacute hemorrhagic presentation from this location has not been previously documented. While intratumoral or subarachnoid hemorrhage has been reported in other cranial nerve schwannomas-including vestibular and trochlear nerves [3,4]-such an event involving the GSPN is exceedingly rare. Proposed mechanisms of hemorrhage in these tumors include vascular ectasia, spontaneous thrombosis with subsequent necrosis, venous congestion, and rupture of fragile intratumoral vessels [5].

Hypervascular cranial schwannomas, particularly those arising from the vagus nerve, jugular foramen, and vestibulocochlear nerve, are known to bleed profusely and pose significant intraoperative challenges. In this context, both elective embolization and emergent embolization have been shown to reduce intraoperative blood loss and enhance the safety of surgical resection. Xu et al. reported successful preoperative embolization of a highly vascular vagal schwannoma using particulate agents, enabling complete and safe resection [11]. Similarly, Rao et al. emphasized the role of angiographic evaluation and embolization in managing hemorrhagic or hypervascular vestibular schwannomas [12]. Our case adds to this body of evidence by extending the utility of preoperative embolization to the GSPN, demonstrating that super selective embolization can achieve immediate hemostasis, alleviate symptoms, and facilitate safe transition to elective microsurgical excision.

A systematic review by Woo et al. further underscores the clinical relevance of timely intervention, noting increased morbidity and mortality associated with hemorrhagic vestibular schwannomas compared to their non-hemorrhagic counterparts [13]. Moreover, delayed hemorrhage has been reported following stereotactic radiosurgery for facial and jugular foramen schwannomas, implicating potential radiation-induced vascular fragility [14,15]. Although Ishikawa et al. described a large cystic GSPN schwannoma causing remote neurological deficits, no hemorrhagic component was identified in that case [16].

Role of embolization 

This case also emphasizes the importance of considering hemorrhagic tumors in the differential diagnosis of acute extra-axial temporal hemorrhages. Other potential entities include meningiomas with hemorrhagic degeneration, hemorrhagic metastases (e.g., melanoma and choriocarcinoma), cavernous hemangiomas, and hemangiopericytomas. In this patient, the sudden onset of right temporal headache with associated vomiting, in the absence of trauma or anticoagulant use, and the identification of a hemorrhagic lesion with intense enhancement and vascular supply from the petrous branch of the MMA prompted urgent consideration of active intratumoral bleeding. The lesion's imaging characteristics, including perilesional edema and contrast accumulation on delayed CT, raised suspicion of ongoing hemorrhage and justified emergent digital subtraction angiography (DSA) to confirm vascular status and enable therapeutic embolization.

A particle size of 150-250 µm was selected to achieve devascularization of the tumor while minimizing the risk of non-target embolization. This choice was based on the anatomical consideration that the petrous branch of the MMA gives off fine perforators to the GSPN and the geniculate ganglion. Smaller particles (<150 µm) could potentially reflux into these delicate perineural vessels, risking ischemic injury and iatrogenic facial nerve palsy. Hence, particle size selection, combined with meticulous catheter positioning and slow, controlled infusion, prioritized both efficacy and safety.

Diagnostic constraints 

Importantly, histopathological confirmation was not obtained in our case, as the patient declined surgical excision following clinical improvement. However, the diagnosis of a hemorrhagic GSPN schwannoma was supported by characteristic radiological features, including a well-circumscribed extra-axial hemorrhagic lesion located at the GSPN hiatus, osseous remodeling of the GSPN hiatus on CT, exclusive vascular supply from the petrous branch of the MMA, and the absence of systemic malignancy or alternative lesions on systemic workup.

While microsurgical resection remains the standard of care for symptomatic or enlarging cranial nerve schwannomas, its role in the acute hemorrhagic setting must be weighed against procedural risk. In our case, the presence of active extravasation from a highly vascular tumor, along with the absence of neurological deficits, favored an initial endovascular approach. Although embolization is typically employed as a preoperative adjunct to reduce intraoperative blood loss, in this instance, it served as an effective temporizing strategy. The patient opted to defer surgery following the resolution of symptoms and continued under clinical and imaging surveillance.

Thus, this case fills a notable gap in the literature by presenting the first reported instance of a hemorrhagic GSPN schwannoma managed successfully with endovascular embolization. While the patient remained neurologically intact and symptom-free at the three-month follow-up, longer-term surveillance is needed to evaluate for recurrence or delayed neurological complications. Continued neurosurgical follow-up has been advised. While embolization alone may provide durable control in select cases, particularly when surgical morbidity is high or the patient declines resection, we acknowledge that its role as a definitive treatment remains investigational and warrants further long-term data.

## Conclusions

Although rare, hemorrhagic GSPN schwannoma should be considered in the differential diagnosis of rapidly symptomatic, enhancing extra-axial lesions in the middle cranial fossa. In the absence of histopathology, characteristic imaging features and vascular correlation can support diagnosis. This case is the first documented instance of a hemorrhagic GSPN schwannoma, expanding the known clinical spectrum of cranial nerve tumors. TAE may serve as a valuable early intervention for hemostasis and even as a definitive therapy in stable patients. A multidisciplinary approach with MRI, CT angiography (CTA), and DSA is essential to guide treatment decisions and identify candidates for embolization. Long-term follow-up remains crucial to monitor for recurrence or delayed complications. Systematic reporting and pooled analyses of similar cases are needed to develop evidence-based management strategies.
